# The use of innovative and efficient nanocomposite (magnetic graphene oxide) for the reduction on of *Fusarium* mycotoxins in palm kernel cake

**DOI:** 10.1038/s41598-017-12341-3

**Published:** 2017-09-29

**Authors:** A. A. Pirouz, J. Selamat, S. Z. Iqbal, H. Mirhosseini, R. Abedi Karjiban, F. Abu Bakar

**Affiliations:** 1 Food Safety Research Centre (FOSREC), Faculty of Food Science and Technology, Universit Putra Malaysia, 43400 UPM Serdang, Selangor Malaysia; 2 Food Safety and Food Integrity (FOSFI), Institute of Tropical Agriculture and Food Security, UPM, 43400 Serdang, Selangor Malaysia; 3 Department of Food Technology, Faculty of Food Science and Technology, Universiti Putra Malaysia, 43400 UPM Serdang, Selangor, Malaysia; 4 Department of Chemistry, Faculty of Science, Universiti Putra Malaysia, 43400 UPM, Serdang, Selangor, Malaysia

## Abstract

Adsorption plays an important role in the removal of mycotoxins from feedstuffs. The main objective of this study was to investigate the efficacy of using magnetic graphene oxide nanocomposites (MGO) as an adsorbent for the reduction of *Fusarium* mycotoxins in naturally contaminated palm kernel cake (PKC). Liquid chromatography-tandem mass spectrometry (LC-MS/MS) was used to assess the mycotoxins in animal feed. Target mycotoxins included the zearalenone (ZEA), the fumonisins (FB_1_ and FB_2_) and trichothecenes (deoxynivalenol (DON), HT-2 and T-2 toxin). Response surface methodology (RSM) was applied to investigate the effects of time (3–7 h), temperature (30–50 °C) and pH (3–7) on the reduction. The response surface models with (R^2^ = 0.94–0.99) were significantly fitted to predict mycotoxins in contaminated PKC. Furthermore, the method ensured a satisfactory adjustment of the polynomial regression models with the experimental data except for fumonisin B_1_ and B_2_, which decrease the adsorption of magnetic graphene oxide (MGO). The optimum reduction was performed at pH 6.2 for 5.2 h at of 40.6 °C. Under these optimum conditions, reduced levels of 69.57, 67.28, 57.40 and 37.17%, were achieved for DON, ZEA, HT-2, and T-2, respectively.

## Introduction

Mycotoxins rarely occur as single contaminants in animal feed and food. Many mycotoxigenic fungi can grow and produce toxic metabolites under similar conditions^1^. A common feature of *Fusarium* species is their capacity to produce ZEA, trichothecenes and FB_1_ and FB_2_ mycotoxins^2^. The contamination of foods and feeds with *Fusarium* toxins is a significant global problem. Developing countries, such as India, South Africa, Philippines, and Thailand have reported a high incidence of DON and ZEA, but, high levels of those toxins also have been documented in developed countries^3^. The *Fusarium* mycotoxins are a family of cytotoxic metabolites from various fungal genera including *Fusarium spp*, *Fusarium graminearum*, *Fusarium proliferatum and Fusarium verticillioides* that have a long-chain hydrocarbon unit^4^. Extensive studies have been carried to investigate the adverse effects of *Fusarium* toxins on animal health and productivity. The main toxic effects of these mycotoxins appear to be a primary inhibition of protein synthesis. The severity of these effects in animal production systems depends on the level of mycotoxins present in the feed supply chain, the duration of exposure, the physiological status of the animal and other environmental factors^5^_._

PKC has been commonly used in feed for ruminants, poultry, swine, and fish. It is abundantly produced in a few main regions of the equatorial tropics, including South East Asia, Africa and South America. PKC may be contaminated with mycotoxin due to climatic conditions and geographical location; thus, the incidence and levels of these mycotoxins vary from country to country^6^.

It is difficult to eradicate mycotoxins once they have been produced in food or feed, and their removal is a great challenge for the agricultural and food industries. Numerous strategies, including physical, chemical and biological techniques, have been studied to decontaminate or detoxify mycotoxins in contaminated food and feed. The major drawbacks to the use of chemicals are their ineffectiveness and their destruction of the nutritional value of animal feed^7^. Furthermore, microbial activity can cause undesirable effects in grains, including discoloration, degradation of lipids and proteins and altered digestibility^8^. The goal of adding adsorption agents to the feed of livestock and poultry is to alleviate the harmful effects of mycotoxins by preventing passage into animal blood and organs via complex formation between these binders and the mycotoxins^9^. Studies have demonstrated the efficiencies of adsorbents with mineral origins hydrated sodium calcium aluminosilicate (HSCAS)^10^, activated carbon^11^, sodium bentonite^12^ and zeolites^13^ and modified materials Montmorillonites treated with organic cations^14^, chitosan polymers^15^ and yeast^16^. However, these adsorbents have been shown to be effective against one or two specific mycotoxin, and are relatively expensive. The MGO nanocomposite is a new class of materials for uses various multifunctional, lightweight structures^17^. It is synthesized from iron oxide nanostructures and graphene oxide (GO). GO is inexpensive and easily accessible and has numerous oxygen that contain functional groups^18^. Nevertheless, carbonaceous adsorbents such as GO suffer from inconvenient separation; it can be difficult to separate GO based adsorbents in aqueous solutions^19^, and traditional separation methods such as filtration and sedimentation are thus rejected. However, magnetic nanoparticles have been widely used in environment remediation due to their ease of separation from aqueous mixtures^20^. The novelty of this study is the use of MGO nanocomposite as an adsorbent to remove *Fusarium* mycotoxins in animal feed.

## Materials and Method

### Chemicals and Materials

A total of 4 types of PKC were obtained from different local factories from different regions (Kelantan and Shah Alam) in Malaysia. Approximately 1 kg of each blank PKC was randomly collected, passed through quarter sampling to create a representative and thoroughly mixed. Sample was ground to a fine powder and sieved (mesh size 750 mm) before being used for spiked. The sample was spiked with ZEA, DON, HT-2, T-2, FB_1_ and FB_2_ standards at three different concentrations (5.0–100.0 ng/g). Each level of spiking was performed in triplicate. The spiked samples were kept overnight in the dark to allow solvent evaporation. In our preliminary study, the amount of MGO from 50 to 300 mg was studied, but the reduction of mycotoxins after 200 mg was not significant. Hence, according to preliminary study the 200 mg was used for all samples. Analytical pure standards of ZEA, DON, FB_1_ and FB_2_, T-2 toxin and HT-2 were supplied by VICAM (Watertown, MA, USA), ferrous ammonium sulfate [(NH_4_)_2_SO_4_FeSO_4._6H_2_O], ammonium ferric sulfate [NH_4_Fe (SO_4_)_2._12H_2_O], nitrate sodium, sulfuric acid 98%, potassium permanganate and hydrogen peroxide were purchased from Merck (Darmstadt, Germany), Graphite powder was supplied from Sigma-Aldrich (St. Louis, MO, USA), deionized water was obtained from a water purifier (Elga Classic UV MK2; Elga, Marlow, UK), fluted filter papers (24 cm) were supplied by (Watman, Maidstone, Kent, UK) and HPLC grade methanol and formic acid were supplied by Merck (Darmstadt, Germany).

### Characterization

The morphologies of GO and MGO were characterized by field emission scanning electron microscopy (FE-SEM) (LEO 1455 VPSEM, Kensington, UK). Transmission Electron Microscope (TEM) (LEO 912AB MA, USA) was used to take the images. Fourier transform infrared spectroscopy (FTIR) spectra were obtained using a Nicolet 6700 (Thermo Nicolet Corp., Madison, WI) and X-ray diffraction patterns were obtained using an (XRD-6000, (SHIMADZU, Japan).

### Apparatus

A blender (Middleton, MA, USA), micropipettes (Eppendorf, Hamburg, Germany), reciprocal vertical shaker (model RS-1, JieoTech co Gyeonggi-do, Korea), vortex mixer (Stuart scientific, Manchester, UK), centrifuge (Sartorius AG, Goettingen, Germany) and nitrogen gas generator (Verdellc, MA, USA) were used during the experiment.

### LC–MS/MS apparatus and conditions

An Agilent 1290 Infinity UHPLC module LC/MS-MS was equipped with an Agilent 6410 Triple Quad LC/MS (Agilent technologies, Palo Alto, CA, USA). The system consisted of a degasser, column oven, and auto sampler. Separation analyses were performed using a Zorbax Eclipse plus, rapid solution HD, C18 (150 × 2.1 mm, 1.8 μm particle size) column (Agilent technologies, Palo Alto, CA, USA) at 30 °C. The system operated with a heated electrospray interface in positive mode (ESI+) at a capillary voltage of 4 kV and nitrogen was used as the spray gas. The desolation temperature was set at 40 °C. Mycotoxins were analyzed in multi reaction monitoring (MRM) channels. Quantification was carried out using matrix-matched standard calibration. As shown in Table 1 the gradient was used a solvent A methanol (mobile phase A) slightly acidified with solvent B 0.1% formic acid in water (mobile phase B) at a flow-rate of 0.4 mL/min.

**Table 1:** Gradient program of the LC/MS-MS.

Step	Time (min)	Solvent A%	Solvent B%	Flow rate (mL/min)
2	1	75	25	0.4
3	6	25	75	0.4
4	10	0	100	0.4
5	14	0	100	0.4
6	18	100	0	0.4

### Preparation of sorbent

#### Production of GO

Graphene oxide were prepared using a modified Hummers method^18^. The GO was synthesized by vigorously stirring commercial graphite powder (5 g) NaNO_3_ (2.5 g) in concentrated 75 mL sulfuric acid 98% in an ice bath at 0 °C. After stirring, potassium permanganate (15 g) was slowly added. The rate of addition was controlled carefully to avoid a sudden increase in temperature. The mixture was then maintained at 35 °C for 30 min. Distilled water (230 mL) was slowly added to the reaction vessel to keep the temperature below 98 °C for 15 min. The mixture was diluted to 700 mL with the subsequent addition of 2.5 mL hydrogen peroxide (wt. 30%). The resulting mixture was filtered washed three times each with ultrapure water and alcohol and then dried at 60 °C for 12 h in a vacuum oven.

#### Production of MGO

The MGO was produced using a chemical co-precipitation method^20^. Iron (III) oxide (Fe_2_O_3_) nanoparticles (5.8 g) were produced by dissolving ferrous ammonium sulfate in (10.7 g) of ammonium ferric sulfate in 100 mL ultrapure water to form a mixed iron salt solution under oxygen-free conditions. Chemical precipitation was achieved by adding a 75 mL ammonium hydroxide (NH_4_OH) solution (29.6%) drop-wise for a period of 30 min at 25 °C. Black precipitates appeared immediately after the addition of ammonium hydroxide and the precipitated particles exhibited a strong magnetic response. Next, 1 g dry GO was dispersed in 100 mL ultrapure water using ultra-sonication to form a stable suspension. Finally, the MGO solid was collected using a magnet and was washed with ultrapure water and anhydrous ethanol three times each and dried at 60 °C for 12 h in a vacuum oven.

### Adsorption Procedure

All sorption experiments were carried out in 50 mL flasks by adding 200 mg of MGO adsorbent to 5 g of PKC contaminated by mycotoxins at three different concentrations (5.0, 25.0 and 100.0 ng/g) and 20 mL distilled water at specified pH values (3–7), prepared using 0.01 M HCl at controlled temperatures (30–50 °C) and equilibrium times (3–7 h) and a constant shaking speed of 300 rpm. After the adsorption process, MGO was conveniently separated by permanent magnet. The residual concentrations of mycotoxins were measured using LC/MS-MS as previously described^21^. The adsorption amount it is based on the concentration in the aqueous solution before and after adsorption, according the following equation^22^.

1 $${\rm{E}}=({{\rm{C}}}_{0}-{{\rm{C}}}_{{\rm{e}}})/{{\rm{C}}}_{0}/100$$

### Experimental design

The simultaneous effects of three independent variables pH (x_1_), equilibrium time (x_2_) and temperature (x_3_) on the reduction on six mycotoxins (Y_1 –_Y_6_), (namely DON, ZEA, HT-2, T-2 toxin, FB_1_ and FB_2_) were evaluated using the RSM. In Table 3 composite central design (CCD), with 20 experiments was assigned to study the main and combined effects of variables on the reduction of mycotoxins. Independent variable ranges studied were pH (3–7), equilibrium time (3–7 h) and temperature (30–50 °C) as shown in Table 2. The center point was repeated six times to determine the repeatability of the method^23^.

**Table 2:** Levels of independent variables established according to the central composite design (CCD).

Independent Variables	Independent variables level		
	Low	Center	High
pH	3	5	7
Time	3	5	7
Temperature	30	40	50

**Table 3:** Matrix of the central composite design (CCD).

Std Order	Block	Run Order	Pt Type	pH	Time (h)	Temperature °C
1	1	1	1	3	3	30
2	2	1	1	7	7	30
3	3	1	1	7	3	50
4	4	1	1	3	7	50
5*	5	0	1	5	5	40
6*	6	0	1	5	5	40
7	7	1	2	7	3	30
8	8	1	2	3	7	30
9	9	1	2	3	3	50
10	10	1	2	7	7	50
11*	11	0	2	5	5	40
12*	12	0	2	5	5	40
13	13	−1	3	3	5	40
14	14	−1	3	7	5	40
15	15	−1	3	5	3	40
16	16	−1	3	5	7	40
17	17	−1	3	5	5	30
18	18	−1	3	5	5	50
19*	19	0	3	5	5	40
20*	20	0	3	5	5	40

### Statistical analysis

Regression analysis and analysis of variance (ANOVA) were conducted to (i) find a relationship between each response and three independent variables and (ii) fit the regression models to the experimental data aiming at an overall optimal region for all response variables studied^24^. The generalized polynomial model for relating the response to independent variables is given below:

2 $${\rm{Y}}={{\rm{\beta }}}_{0}+\sum {{\rm{\beta }}}_{{\rm{i}}}{{\rm{x}}}_{{\rm{j}}}+\sum {{\rm{\beta }}}_{{\rm{ii}}}{{\rm{x}}}_{{\rm{i}}}^{2}+\sum {{\rm{\beta }}}_{{\rm{ii}}}{{\rm{x}}}_{{\rm{i}}}{{\rm{x}}}_{{\rm{j}}},$$

where Y is the response calculated by the model, β_0_ is a constant and βi, β_ii_ and β_ij_ are linear, quadratic and interaction coefficients, respectively. For a good model fit, the R^2^ should be at least 0.80. The adequacy of the models was determined using model analysis, lack-of fit tests and coefficient of determination (R^2^) analysis as outlined by previous studies^23,25^. The experimental design matrix, optimization procedures and data analysis were achieved using the Minitab V.16 statistical package (Minitab Inc., State College, PA, USA).

### Optimization and validation procedure

Graphical and numerical multiple optimization procedures were carried out using the Minitab software to identify the optimum reduction process variables (i.e. pH, time and temperature), for the desired response goals. In graphical optimization, the reduced response models were described as three-dimensional (3D) surface plots to better visualize the significant (*p* < 0.05) interaction effects of all factors on the response. In numerical response, optimization allowed us to interactively change the input variable settings and perform sensitive analyses to possibly improve upon the initial solution^26^. To verify the adequacy of the regression equations, the experimental data were compared with the predicted values obtained from the equations. There can be no significant (*p* > 0.05) difference between the experimental and predicted values. In this study, multiple graphical and numerical optimization procedures were applied to determine the optimal region of pH, time and temperature for the overall response goals^27^.

## Results and Discussion

### Characterization of GO and MGO

**Figure 1:**
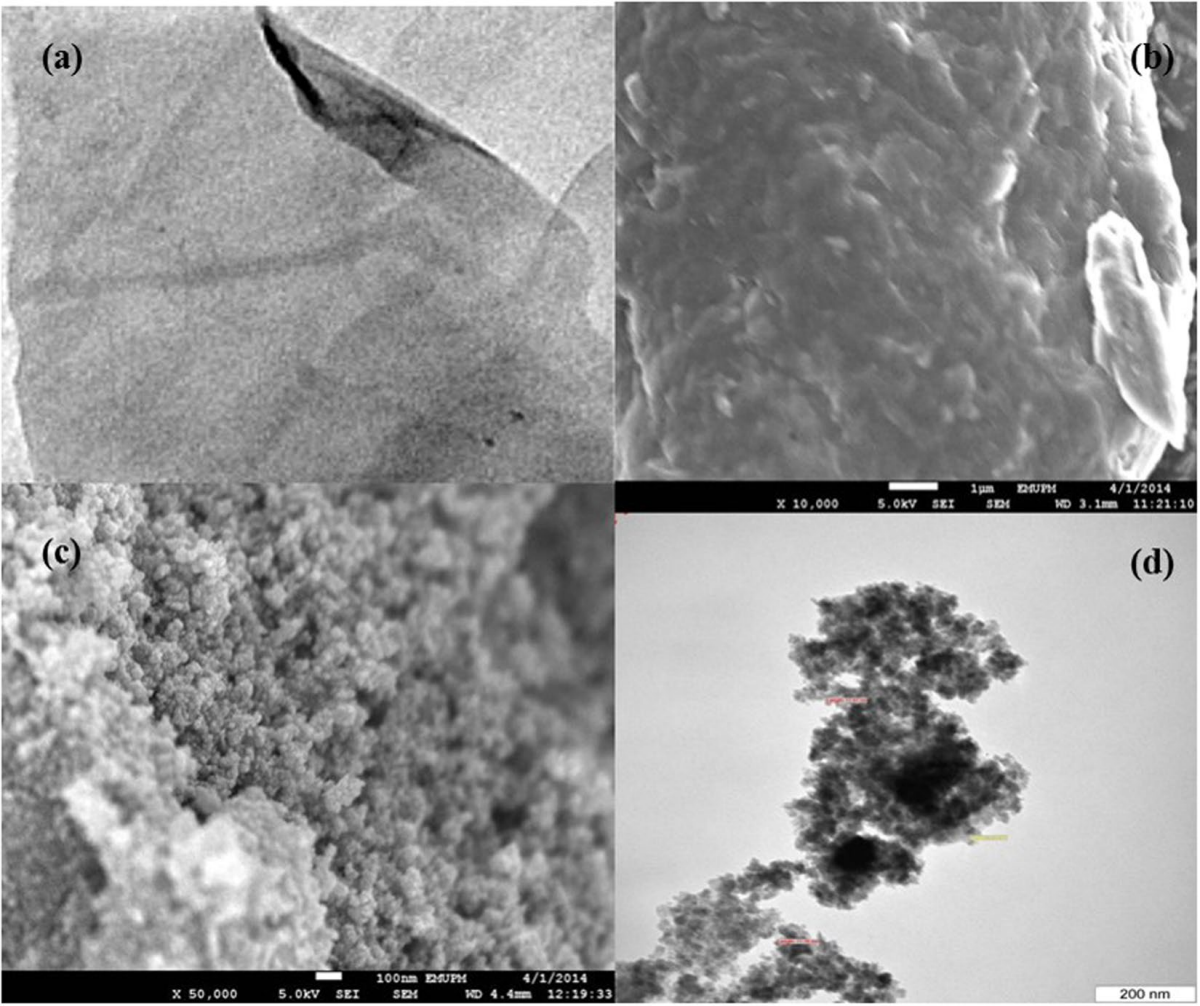
TEM and FESEM images of GO and MGO: (**a**) FESEM of GO, (**b**) FESEM of MGO, (**c**) TEM of GO and (**d**) TEM of MGO.

Figure 1b shows FESEM and TEM: FESEM images of the fracture surfaces of GO and MGO composites after tensile testing. The GO presents a sheet-like structure (Fig. 1a), whereas a rougher surface is presented after combination with Fe_3_O_4_ (Fig. 3b). The MGO mainly consists of carbon-encapsulated Fe with a size of 2–10 nm. TEM images for GO and MGO are presented in Fig. 1(c,d). The image of GO illustrates (Fig. 1c) at smooth surface, wrinkled edges and transparent and wavy structures, which are characteristic of GO sheets^28^ that are chemically deposited on the GO surface of a large fraction of Fe_3_O_4_ particles. The mean diameter of the Fe_3_O_4_ nanoparticles on GO is 8–10 nm with a narrow size distribution. Some Fe_3_O_4_ aggregation is observed in Fig. 1d ^29^^.^

**Figure 2:**
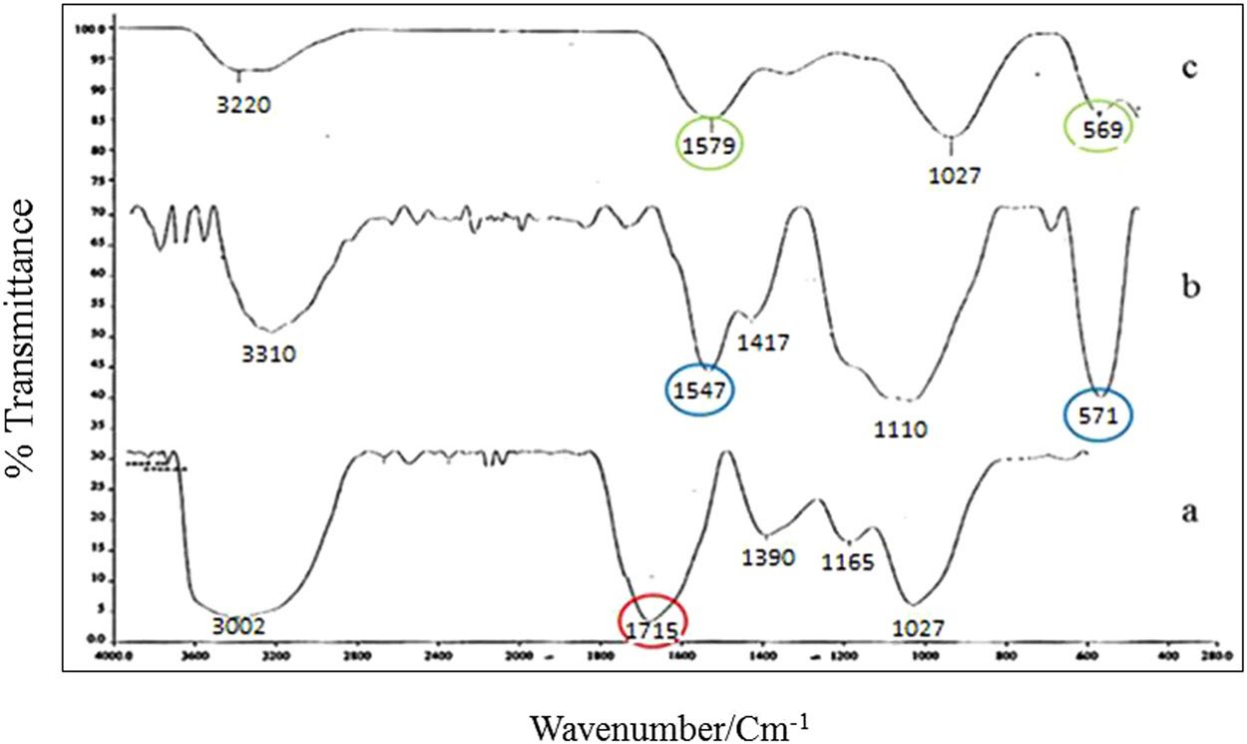
FTIR spectra of (**a**) GO, (**b**) MGO, (**c**) mycotoxins-loaded magnetic graphene oxide.

The FTIR is a valuable technique for understanding the mechanism of composite formation and/or mycotoxins adsorption. The GO and MGO had been characterized by FTIR (Fig. 2). The GO exhibits the presence of the oxygen containing functional groups such as hydroxyl, epoxy and carboxyl groups^30^. The characteristic spectrum at 1715 cm^−1^ corresponds to C–O–C stretching vibrations, O–H deformation of C–OH groups, the C=C stretching mode of the sp^2^ C network and the C=O stretching vibrations of the COOH group, respectively (Fig. 2a)^22^. The peaks located at 1547 cm^−1^ and 571 cm^−1^ correspond to epoxy groups, complying with symmetric stretching and Fe_3_O_4_ nanoparticles. The peak at 571 cm^−1^ is ascribed to the Fe-O group (Fig. 2b). The peak at 1715 cm^−1^ which corresponding to the C=O of carboxyl group on the GO shifts to 1547 cm^−1^ possibly due to the formation of –COO- after coating with Fe_3_O_4_^31^. FTIR technique therefore suggest that the removal mechanism of mycotoxins reduction by MGO as an adsorbent involves two steps (Fig. 2c). The π–π stacking is predominantly based on the π-π interaction between the aromatic ring of mycotoxins and the GO basal planes. Moreover, electrostatic attraction between the negatively charged analytes (COO^−^) and the positively charged iron ions (Fe^+2^ and Fe^+3^) of MGO accelerated the electron transfer between the materials.

The XRD patterns of GO, and MGO composite are presented in Fig. 3. Because GO prepared from graphite by liquid-phase oxidation is an intercalation compound, the d- spacing of GO layers is 2θ = 38.4°. The MGO primarily consists of a mixture of iron oxide and GO, where intensity was calculated at 2θ = 44.6°, 58.9° and 60.2° consistent with the standard XRD data for the cubic phase of Fe_3_O_4_^32^. The disappearance of the GO peak in the XRD pattern of MGO may be because (1) more monolayer graphene is caused the reduction of graphene sheet aggregation in the presence of magnetite, resulting in weaker peaks from carbon or (2) the strong signals of the iron oxides overwhelm the weak carbon peaks^19^.

**Figuree 3:**
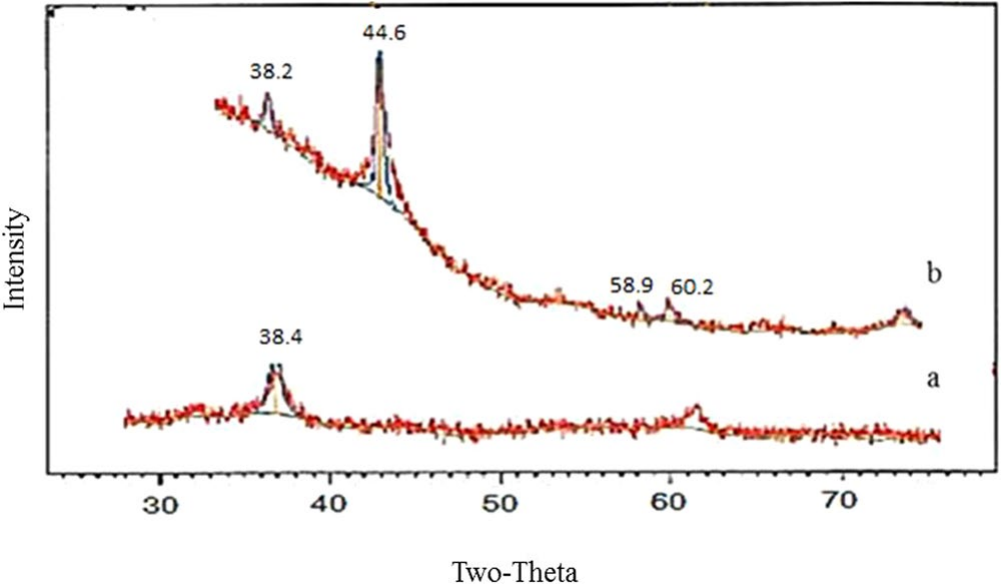
XRD patterns of (**a**) GO, (**b**) MGO.

### Fitting of response surface models to significant independent variables

The estimated regression coefficients of the response surface models, along with the corresponding R^2^, R^2^ (adj), F-value and *p*-value of lack of fit for final reduced models are given in Table 4. The individual significance of independent variables determined using the F-ratio and *p*-value are also shown in Table 5. As shown in Table 4, the significant (*p* < 0.05) response surface models with high R^2^ values, ranged from 0.94 to 0.99, were obtained to evaluate the reduction of mycotoxins, expect FB_1_ and FB_2_ where R^2^ values for these response variables were less than 0.80 (0.78 and 0.67) respectively. This may be due to the aliphatic long-chain molecules of FB_1_ and FB_2_ which are not effective against MGO. As noted, the adsorption of FB_1_ and FB_2_ are dependent on their solubility according to polarity and other characteristics affecting toxin reduction^33,34^. Hence, more than 70% of the response variations can be explained as a reduction in *Fusarium* toxins by MGO.

**Table 4:** Regression coefficients, R^2^, adjusted R^2^, probability values and lack of fit for the response resurface model.

	DON	ZEA	HT-2	T-2	FB1	FB2
b_0_	69.20	69.76	49.55	27.06	—	—
b_1_	10.00	−5.37	9.92	4.30	—	—
b_2_	−11.38	1.41	−6.69	5.14	—	—
b_3_	−8.30	4.43	11.55	0.55	—	—
b_1_^2^	−8.38	—	3.26	18.20	—	—
b_2_^2^	−20.85	5.32	—	—	—	—
b_3_^2^	—	−4.54	−7.41	−18.94	—	—
b_12_	—	2.83	—	4.22	—	—
b_13_	−10.59	—	3.54		—	—
b_23_	6.69	1.56	2.03	6.65	—	—
R^2^	0.97	0.94	0.99	0.97	0.87	0.67
Regression (*p*-value)	0.00	0.00	0.04	0.01	0.29	0.05
Lack of fit (F-value)	1.22	24.00	3.50	6.91	—	—
Lack of fit (p-value)	0.43	0.00	0.17	0.07	—	—

**Table 5:** The significance of each independent variable effect using F-ratios an *p*-values in the final reduced models.

Response		Linear effects			Quadratic effects			Interaction effects		
		X_1_	X_2_	X_3_	X_1_^2^	X_2_^2^	X_3_^2^	X_1_X_2_	X_1_X_3_	X_2_X_3_
DON(Y_1_)	*p*-value	0.000	0.000	0.000	0.009	0.000	—	—	0.000	0.002
	F-Value	43.66	56.42	30.00	9.79	60.59	—	—	39.07	15.57
ZEA(Y_2_)	*p*-value	0.00	0.038	0.001	—	0.00	0.001	0.000	—	0.040
	F-Value	79.07	5.47	53.77	—	24.84	18.09	17.54	—	5.30
HT-2(Y_3_)	*p*-value	0.00	0.220*	0.007	0.007	—	0.001	—	0.000	0.006
	F-Value	355.58	1.71	482.43	11.68	—	60.37	—	37.36	11.94
T-2(Y_4_)	*p*-value	0.00	0.00	0.00	0.000	—	0.000	0.001	—	0.000
	F-Value	29.46	41.95	0.47	160.02	—	173.12	22.68	—	56.22

X_1_, X_2_ and X_3_ represent the main effects of pH, time and temperature X_1_^2^, X_2_^2^ and X_3_^2^ represent the quadratic effects of pH, time and temperature X_1_X_2_, X_1_X_3_, X_2_X_3_ represent the interaction between pH and time, pH and temperature and time and temperature.

*Not significant at *p* > 0.05.

The results show a significant lack of fit (*p* < 0.05) for the model fitted for ZEA. A model with a significant lack of fit could still be used when large amounts of data are included in the analysis^35^. Therefore, the results ensured the satisfactory fitness of the polynomial regression models with the experimental data in Table 5.

### Relationship between independent variables on reduction of multi-mycotoxins

**Figure 4:**
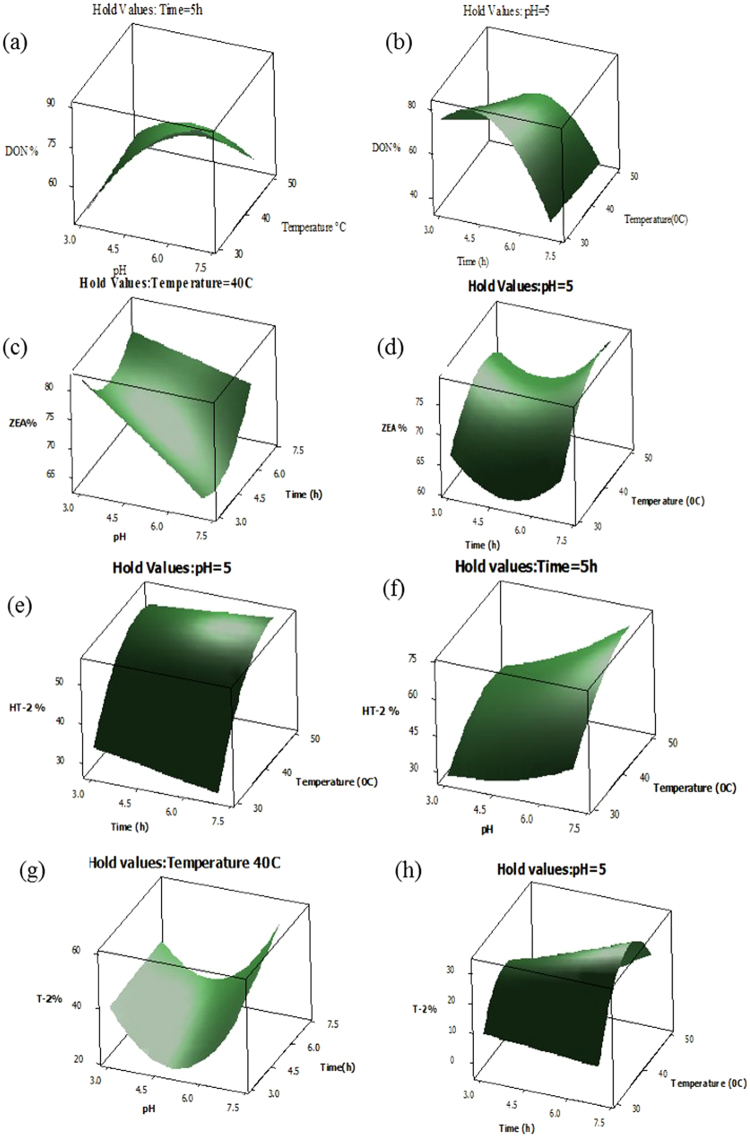
Response surface plots showing the significant (*p* < 0.05) interaction effects of reduction of DON (**a**,**b**), ZEA (**c**,**d**), HT-2 (**e**,**f**) and T-2 (**g**,**h**).

Table 5 illustrates F and *p*-values for the main, quadratic effect and interaction of each variable (pH, time and temperature). From the results, most variables showed significant reduction effects (*p* < 0.05) for mycotoxins. The main linear effects of all variables had more significant (*p* < 0.05) effect on mycotoxin reduction compared to their quadratic and interaction effects. The effect of pH was greater than those of time and temperature as a main effect (Table 5). As shown in Table 5, all variables had significant reduction (*p* < 0.05) effects on the mycotoxins and should therefore be kept as critical parameters in the final reduced model fitted to the experimental data. The quadratic term of pH and temperature for T-2 illustrated the highest effect on the reduction; the F-values were 160.02 and 173.2 for pH and temperature, respectability. The interaction effects of pH with two other factors significantly (*p* < 0.05) influenced the reduction of mycotoxins. The results show that the interaction effects of pH and time were significant (*p* < 0.05) for some response variables, including ZEA and T-2. Furthermore, this type of interaction, according to Table 4, also has positive effects. Although the interaction between pH and temperature had significant effects on the reduction of DON and HT-2, the reduction of these mycotoxins depended on temperature at equilibrium. Table 4 indicates that the interaction had as negative effect for DON and a positive effect for HT-2. According to Table 5, the interaction term of time and temperature had reducing effect on all mycotoxins. Figure 4 presents 3D surface plots including the significant interaction effect of processing variables on the reduction of various mycotoxins. Figure 4a,b,e, and f illustrated the effects of pH with temperature and time with temperature on the percent of reduction of DON and HT-2, respectively. The interaction of pH and temperature as well as time and temperature, had significant (*p* < 0.05) effects on the reduction value of ZEA and T-2 respectively (Fig. 4b,c,g, and h). This confirmed that the reduction of all targets significantly (*p* < 0.05) depended on processing temperature and equilibrium time.

### Optimization and procedure for predicting the highest

Numerical and graphical multiple response optimizations were performed for determining the optimum levels of independent variables to achieve the desired response goals. The numerical multiple optimizations results showed that the overall optimum point for maximum simultaneous reduction of multi-mycotoxins with total desirability (D = 0.623) was predicted to be 6.2 of pH, 5.2 h and 40.6 °C. Under these optimum conditions, the response variables for DON, ZEA, HT-2 and T-2 were predicted to be 69.57, 67.28, 57.40 and 37.17%, respectively. Graphical optimization procedures were used to investigate the effect of independent variables on the response variable. Three-dimensional response surface graphs were used to illustrate the effect of variables on the % reduction of *Fusarium* toxins. The adequacy of the response surface equations was confirmed by comparing the experimental and fitted values predicted by the response regression models. No significant difference was found between these values. The experimental response values were shown to be in agreement with the predicted values.

### Verification of models

The verification experiment was performed by comparing the experimental and predicted fitted values, which are presented in Table 6. No significant (*p* < 0.05) difference was reported between the experimental and predicted values (test value) and there was a close correspondence between these values. Therefore, predicted values verified the adequacy of the models fitted by RSM.

**Table 6:** Experimental and predicted values for the response variables.

Response	pH	Time	Tem	Y_0_	Y_i_	Y_0_-Y_i_	Desirability
DON	6.2	5.2	40.6	68.92	69.57	0.35	0.98
ZEA	6.2	5.2	40.6	66.02	67.28	−1.26	0.62
HT-2	6.2	5.2	40.6	59.78	57.40	2.38	0.68
T-2	6.2	5.2	40.6	38.95	37.17	1.78	0.35

Y_0_: predicted value; Y_i_: experimental value; Y_0_-Y_i_: residue.

## Conclusion

This is the first time that, MGO absorption of *Fusarium* species has been investigated simultaneously in PKC as an ingredient of animal feed. Statistical analysis using RSM with CCD was shown to be a robust experimental design for optimizing the effects of pH, time and temperature to explain the variation of six response variables. Analysis of variance (ANOVA) illustrated a high coefficient of determination value R^2^ (0.94–0.99) and a non-significant (*p* > 0.05) lack of fit, ensuring a satisfactory adjustment of the second-order regression model with the experimental data. The response surface and graphical optimization methods lead to a better understanding for optimizing the clarification process. The best condition for reduction of mycotoxins was 6.2 pH for 5.2 h at 40.6 °C. The reduction of mycotoxins was found to vary from 37.17% to 69.57% for T-2 and DON, respectively. As shown, the lowest reduction was for T-2 because of its moderate hydrophobicity T-2 is a non-ionizable molecule with a bulky oxygen group that does not favor adsorption to planar surfaces. In addition, it adsorbs very little with MGO. The present method offers clear advantages, including simplicity and a reduced low concentration of adverse mycotoxins in the sample. This is important for all separations of adsorbent because mycotoxins were diminished more than by other methods. In addition, MGO is relatively inexpensive and it is quite satisfactory with respect to some mycotoxins simultaneously therefore, it would be good adsorbent for practical applications.
